# Quantifying the impact of the COVID-19 pandemic on clinical trial screening rates over time in 37 countries

**DOI:** 10.1186/s13063-023-07277-1

**Published:** 2023-04-04

**Authors:** Kelsey McDonald, Earl Seltzer, Mary Lu, Stefan Diaz Gaisenband, Cassandra Fletcher, Patrick McLeroth, Kamal S Saini

**Affiliations:** 1 Labcorp Drug Development Inc, 206 Carnegie Center Dr, Princeton, NJ 08540 USA; 2 Addenbrooke’s Hospital, Cambridge University Hospitals NHS Foundation Trust, Cambridge, UK

**Keywords:** COVID-19, Coronavirus, Pandemic, Clinical trials, Screening, Enrollment, SARS-CoV-2, Global, Impact, Recruitment

## Abstract

The COVID-19 pandemic has had an unprecedented and disruptive impact on people’s health and lives worldwide. In addition to burdening people’s health in the short-term in the form of infection, illness, and mortality, there has been an enormous negative impact on clinical research. Clinical trials experienced challenges in ensuring patient safety and enrolling new patients throughout the pandemic. Here, we investigate and quantify the negative impact that the COVID-19 pandemic has industry-sponsored clinical trials, both in the USA and worldwide. We find a negative correlation between the severity of the COVID-19 pandemic and clinical trial screening rate, with the relationship being strongest during the first three months of the pandemic compared to the entire duration of the pandemic. This negative statistical relationship holds across therapeutic areas, across states in the USA despite the heterogeneity of responses at the state-level, and across countries. This work has significant implications for the management of clinical trials worldwide in response to the fluctuating severity of COVID-19 moving forward and for future pandemics.

## Introduction

On December 31, 2019, a cluster of pneumonia cases associated with a novel coronavirus, SARS-CoV-2 (hereafter COVID-19), was first reported from Wuhan, China. Following identification, a steep rise in reported confirmed cases across the world resulted in the World Health Organization (WHO) declaring the COVID-19 outbreak a global pandemic on March 11, 2020. As of this writing, more than 669 million confirmed cases of SARS-CoV-2 infection have been reported globally, and 6.74 million people have died worldwide due to COVID-19, including 1.02 million in the USA alone [1].

Since the onset of the pandemic, there has been considerable heterogeneity at the country, regional, and even municipal level in terms of the level of preparedness and response to this global threat [2, 3]. Countries have generally followed a combination approach of containment and mitigation activities with the intention of preventing a spike in hospitalization and overwhelming the local healthcare infrastructure. Not surprisingly, in parallel to the public health impacts, COVID-19 has also greatly disrupted human clinical research activities over the first 24 months of the pandemic [4, 5].

Using data from a large central laboratory that supports clinical trials globally, we have evaluated the relationship between COVID-19 pandemic severity and clinical trial screening both within the USA and around the world. We focus on the impact of COVID-19 on clinical trial activity from the onset of the outbreak and demonstrate how the subsequent changes in the distribution and severity of the pandemic relate to clinical trial screening over time. This work contributes to ongoing needs of medical product development companies, sponsor organizations, and public health entities to better quantify the impact of the pandemic and model future trends.

## Methods

Our aim was to quantify the extent to which COVID-19 impacted industry sponsored clinical trial screening rates across a range of therapeutic indications, both in the USA as well as globally. We define the weekly screening rate of a clinical trial in a particular state (USA) or country as the total number of patients screened by all sites within a study protocol in that geographic region divided by the average number of open clinical sites for that protocol in that region that week. We define patients as screened at the first clinical study visit for a clinical trial participant where trial eligibility is formally evaluated and after informed consent is signed. This is most often different from screening or randomization where a trial participant is assigned to a specific intervention as prescribed in the design of the research study. Screening rates were chosen instead of enrollment or randomization because, at our central laboratories, initial screening kits are a robust measure of clinical trial enrollment. Additionally, the pandemic affected clinical trial research in a holistic and all-encompassing manner; therefore, we did not wish to allow for specific trial eligibility attributes to confound the analysis.

For quantifying the magnitude of the COVID-19 pandemic, we utilize the time-series data of COVID-19 cases and deaths made publicly available on the COVID-19 Dashboard by the Center for Systems Science and Engineering (CSSE) at Johns Hopkins University (JHU) (https://systems.jhu.edu/research/public-health/ncov/). [1]. This dataset is a composition of multiple official data sources on the COVID-19 pandemic, such as the US Center for Disease Control and Prevention (US CDC), World Health Organization (WHO), and the European Centre for Disease Prevention and Control (ECDC), among others (https://github.com/CSSEGISandData/COVID-19). Trial screening information originated from Labcorp clinical trial central laboratory where the number of screening (or first participant) lab kits by study over time was used as a proxy for clinical trial recruitment. Because most industry sponsored clinical trials require a baseline sample of participant laboratory characteristics for eligibility determination, observing the rates at which these baseline kits were received by the lab for processing allowed us to approximate trial activity over time. Our analysis time period is from the onset of the COVID-19 pandemic in a given country (approximately early 2020) up to 28 March 2022. For pre-pandemic comparisons, data from 2019 was used as a baseline.

From our central laboratory database as described above, we applied a set of filtering criteria in order to investigate the impact COVID-19 had on our ongoing trial accrual. Because we needed to assess the actual impact of the pandemic on trials, it was important that studies included were initiated well before the pandemic and continued through at least the first year of the pandemic. Such study attributes allow us to establish a stable baseline screening against which any changes during the pandemic period could be compared. Specifically, we included clinical trial protocols only if they

- 1) Were initiated (i.e., the first day a kit was delivered to a site as a proxy for study start) before or on January 30, 2019
- 2) Had the last patient’s first visit date on or after March 30, 2021 (the first full year of the pandemic), to allow for at least 12 months’ worth of data to analyze
- 3) A study’s initial laboratory activity occurs before or on the same date as the first patient first visit date

We also only include studies in clinical phase 2, 2a, 2b, 3, 3a, or 3b from the following therapeutic areas: cardiovascular, central nervous system (CNS), metabolic/endocrinology, infectious disease (excluding any clinical trials relating to COVID-19), oncology, and autoimmune/inflammation. COVID-19 trials, both therapeutic and prophylactic, were excluded because they were designed to enroll patients affected by the pandemic. Phase 1 and 4 trials were excluded because these studies often have design characteristics that would impact our ability to compare screening from one study to another (e.g., cohort based designs in phase 1 trials that do not enroll linearly). Other indications were not included due to comparatively smaller number of such trials and could affect our ability to maintain client confidentiality in our analysis. After applying these criteria, we had 221 clinical trial protocols included in our clinical trial screening dataset for analysis.

The results section is organized as follows: first, we analyze the impact of COVID-19 by looking at the year-over-year % difference (YoYD) in screening rate to compare clinical trial screening rate after the onset of the pandemic to the same time last year. Next, we perform correlational analyses between COVID-19 pandemic severity and screening rate within the USA, with a particular focus on the state-by-state heterogeneity. In our initial dataset, the median number of unique trial protocols per country was 19. We restricted our subsequent analyses to the 37 countries with at least 19 trial protocols, to build an understanding of how the COVID-19 pandemic has impacted clinical trials around the world.

## Results

### Profiling COVID-19 impact by year-over-year difference

**Fig. 1:**
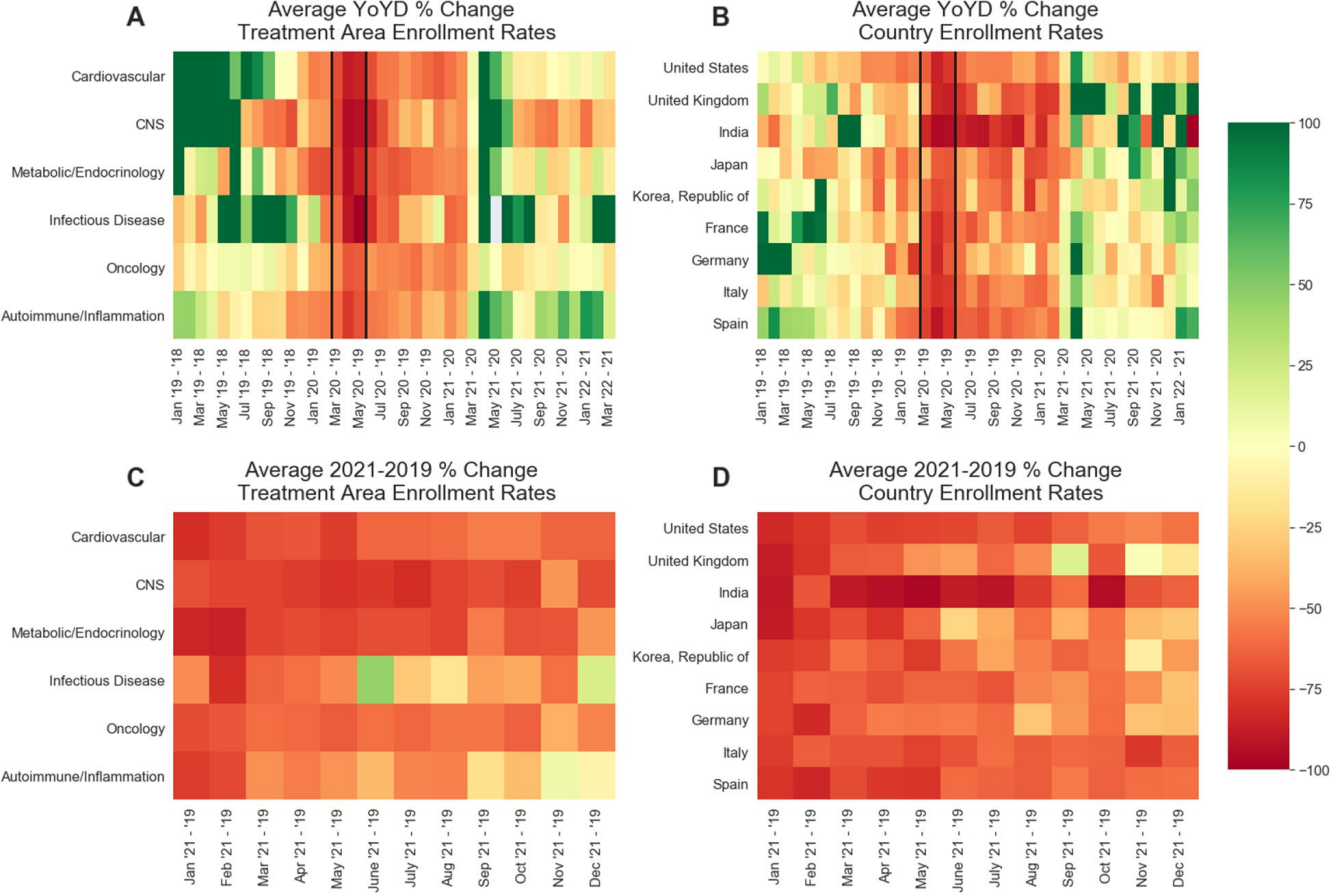
Heatmap illustrating the average year-over-year difference (YoYD) percentage in clinical trial site screening rates. **A** Average YoYD change (%) in six major treatment areas: cardiovascular, central nervous system (CNS), metabolic/endocrinology, infectious disease, oncology, and autoimmune/inflammation. The 3-month period from March to May 2020 (marked in vertical black lines) observed the largest decline across therapeutic areas, with an average of 77.3% across the selected treatment areas. We observe positive YoYD % changes starting around April 2021 compared to April 2020, which is primarily due to the heavily decreased enrollment rates in April of 2020. **B** Average YoYD change (%) among nine major representative countries in our clinical trial sample. We observed an average of 73.8% decrease in enrollment rates from March to May 2020 compared to those months in 2019. **C** Comparison of YoYD from 2021 compared to 2019 for treatment areas and **D** major representative countries in our sample. While we observe sustained decreases in 2021 enrollment rate compared to pre-pandemic levels, we note that the decline is lessening over time, and some treatment areas are observing a rebounding of enrollment rates to pre-pandemic levels, such as in infectious disease trials and autoimmune/inflammation clinical trials

First, we examined the YoYD in clinical trial screening rate for the protocols included in our dataset. This compares the screening rate for a given time period in a particular year to the same time period from the previous year (for instance, we calculate YoYD (%) in screening rates for 2020 compared to those in 2019 by subtracting the screening rate for 2020 from the screening rate for 2019 and then dividing by the screening rate for 2019 to obtain the percentage change. Averaged across therapeutic areas, we observed a 76.8% decline in the average rate of new patients entering trials per study-site year-over-year from end of March to end of May 2020 (noted in vertical black lines in Fig. 1A, B) compared to the same time frame in 2019. In addition, within a subset of countries visualized in Fig. 1B (India, Japan, South Korea, France, Germany, Italy, Spain, US, and UK), we observe a 73.3% decline in screening rate from March to May in 2020 compared to March to May in 2019.

While the YoYD in clinical trial screening rate comparing 2020 to 2019 quantifies the decline in screening rate during the onset of the COVID-19 pandemic, we can use the difference from 2021 to 2019 as a metric for quantifying whether a given therapeutic area or country was able to return to pre-pandemic screening rate levels after the first year; see Fig. 1C/D. We observe an overall decline in average clinical trial screening rates in 2021 compared to the same time frame in 2019, indicating the global pandemic was still negatively impacting clinical trial operations. This 2021–2019 decline varied by treatment area, with infectious disease showing the least decline (31.5%) and CNS demonstrating the largest decline (65.6%). We also observe the UK’s screening rates starting to return to pre-pandemic levels in September 2021 (compared to those in September 2019). Of the representative countries visualized in Fig. 1D, the country with the least average decline in 2021 compared to pre-pandemic (2019) screening rate levels was the UK with 42.2% decline, and the country with the most decline was India with 77.9% decline. Finally, the magnitude of decline in the second half of the 2021–2019 time frame is less than that of the first half, suggesting that screening rates are improving, compared to the period of maximal impact in spring 2020, as the time from the onset of the COVID-19 pandemic increases.

### Relationship between COVID-19 severity and screening rate within the USA

The preceding YoYD analysis profiles clinical trial screening rates over time, with a particular focus on the March 2020 onset of the COVID-19 pandemic in the USA. In order to determine the extent to which metrics quantifying the severity of the COVID-19 pandemic over time correlate to screening rate activity, we assessed the Pearson correlation coefficient $r$ between weekly clinical trial screening rates and the weekly total number of new COVID-19 cases or deaths according to the Johns Hopkins COVID-19 data in the previous week for each country as well as US state included in our dataset. We lag the COVID-19 metrics back by 1 week for all correlational analyses in this manuscript unless stated otherwise because we hypothesized (and observed) that negative impacts on screening rate would occur as a result of changing COVID-19 conditions.

**Fig. 2:**
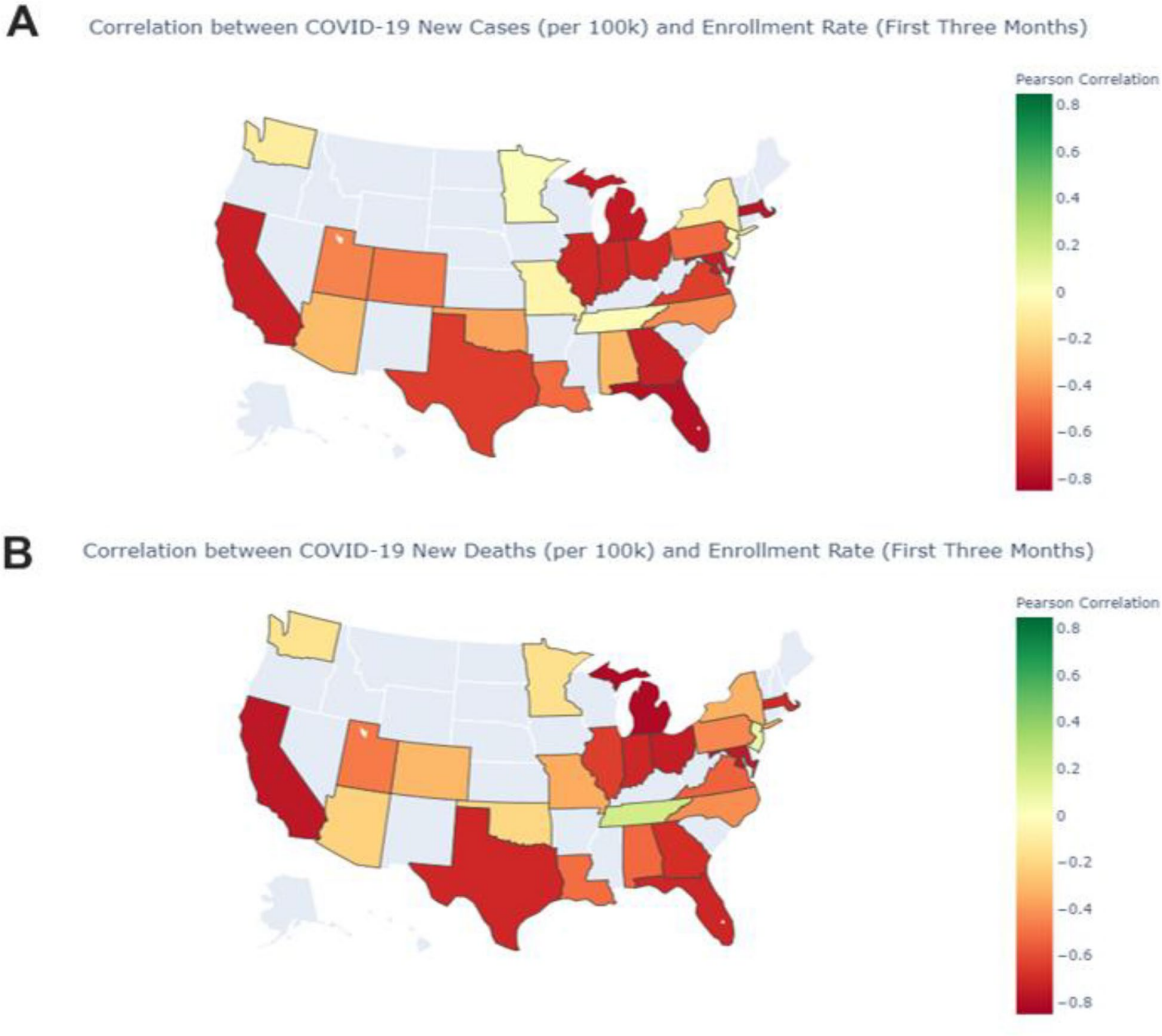
Pearson correlation between clinical trial enrollment rates and total weekly COVID-19 **A** cases and **B** deaths in the USA. Gray states were not included in the analysis due to small sample size of trial protocols; only the top 50th percentile of states (i.e., states with at least 27 unique clinical trial protocols ongoing in 2019) are displayed in color. The color bar notes the Pearson correlation, with green values indicating positive correlations and red values denoting negative correlations between COVID-19 severity and enrollment rates

Due to the decentralized, heterogeneous response to the COVID-19 pandemic within the USA [3, 6], we first observe the correlation between COVID-19 pandemic severity and screening rate at the US state level. We analyzed only the US states that were in the top 50th percentile of number of unique clinical trial protocols in 2019 (median threshold = 27 unique trials), due to small numbers of clinical trial protocols in some states. Overall, the COVID-19 pandemic had a large, negative impact on clinical trial screening rates at the state level in the USA. Figure 2 shows the Pearson *r* correlation between the first 12 weeks of the COVID-19 pandemic (marked as the first week in which a COVID-19 case was confirmed in that state) and the following week’s screening rate for that state. All of the states with a significant Pearson correlation at the *p* < 0.05 level between either COVID-19 cases or deaths (per capita) and screening rate were negatively correlated, indicating that the severity of the pandemic negatively impacted screening rates, with the most severe impact occurring during the first 3 months of the pandemic. Only four states had a correlation above zero with either COVID-19 cases (New York, *R* = 0.10), deaths (Tennessee, *R* = 0.15), or both (New Jersey, cases *R* = 0.0014, deaths = 0.14; Washington, cases *R* = 0.01, deaths *R* = 0.0007), all of which were non-significant findings. The average correlation across every state included in our sample for the first 12 weeks of the pandemic for COVID-19 cases is *r* = − 0.459 and for deaths is *r* = − 0.464.

**Fig. 3:**
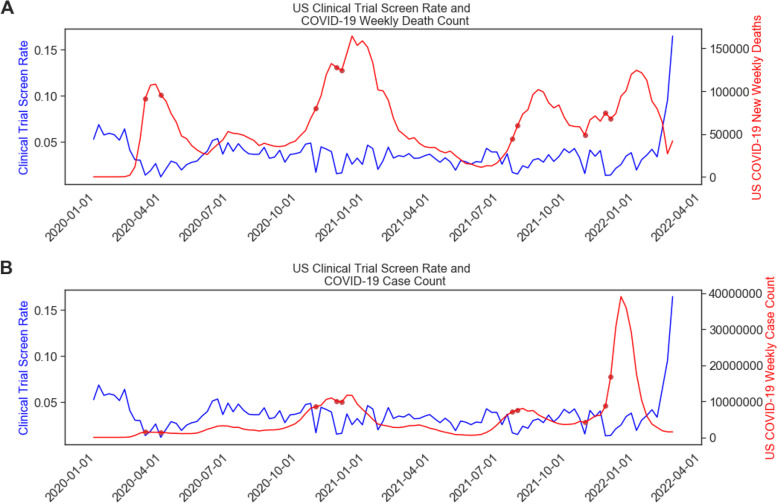
Relationship between COVID-19 death and case counts and clinical trial enrollment rate in the USA. Line plot of the weekly clinical trial screening rate since the beginning of 2020 in blue, and the weekly sum of newly reported COVID-19 **A** deaths and **B** cases in the USA. Red scatter points show the ten dates with the lowest enrollment rates in our dataset; these dates correspond to dates with either peaks or rapidly increasing rates of either COVID-19 cases or deaths

Next, we quantified the Pearson correlation between US COVID-19 severity and clinical trial screening rates across the entire USA (see Fig. 3). For the time period from the onset of the first COVID-19 case in the USA (January 22, 2020) to the last date in our clinical screening rate sample (March 28, 2022), we observe a significant, negative correlation between COVID-19 new deaths and clinical screening rate (*r* = − 0.38, *p* < 0.0001). This is less negative than the correlation that exists during just the first 3 months of the pandemic (*R* = − 0.92, *p* < 0.0001), indicating that the strength of the relationship between the pandemic death rate and the clinical trial screening rates has weakened over time. Indeed, the 10 weeks with the lowest clinical site screening rates (marked in Fig. 3 with red points) correspond to times in the pandemic in which either cases or deaths were near a peak (deaths in mid-April 2020, cases in December 2020, cases in August 2021 associated with the delta variant) or near a sharp increase in pandemic severity, such as the spike in cases associated with the omicron variant at the end of 2021/beginning of 2022).

### Global relationship between COVID-19 severity and screening rate

**Fig. 4:**
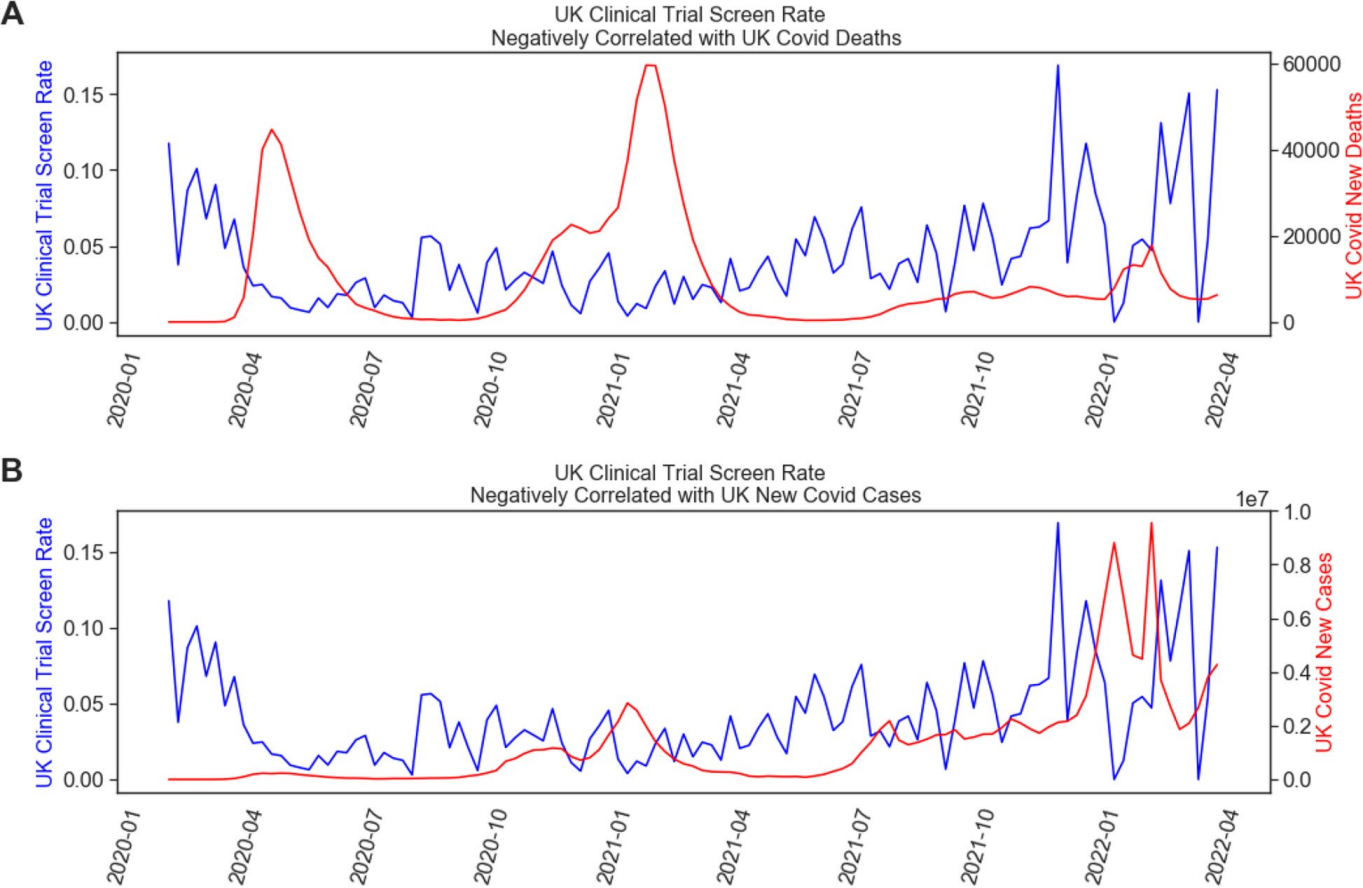
Relationship between COVID-19 pandemic severity and clinical trial enrollment rates in the UK. **A** Line plot of the clinical trial screening rate since the beginning of 2020 in blue, and the weekly total number of newly reported COVID-19 deaths in the UK. **B** Line plot of the screening rate (same as in **A**) overlaid with the weekly total number of newly reported UK COVID-19 cases in red

**Fig. 5:**
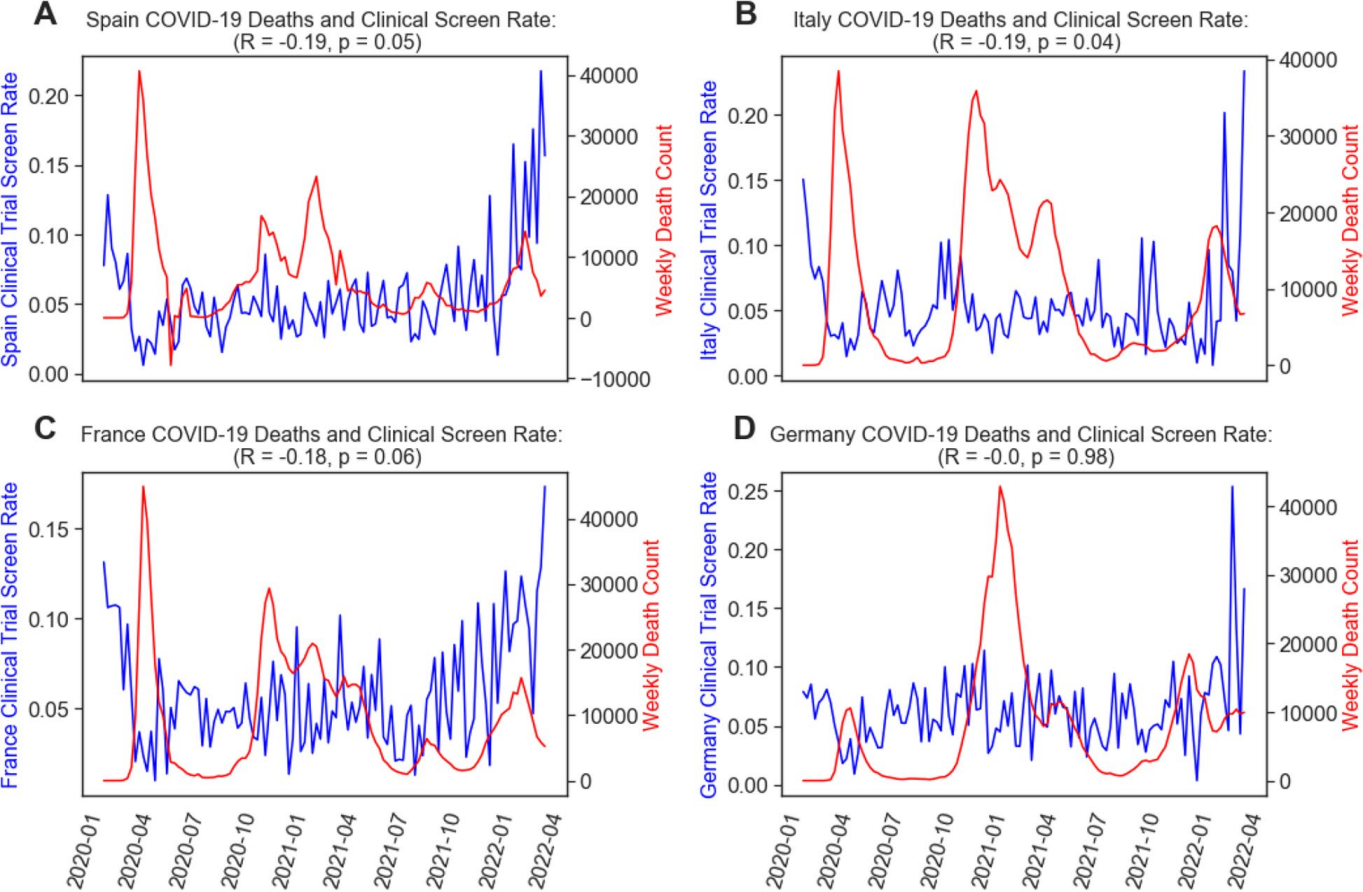
Relationship between COVID-19 pandemic severity and clinical trial enrollment rates in the four largest countries in our enrollment rate sample after the USA: Spain, Italy, France, and Germany. Each subplot is a line plot of the clinical trial screening rate since the beginning of 2020 in blue and the weekly total number of newly reported COVID-19 deaths in **A** Spain, **B** Italy, **C** France, and **D** Germany. Title includes the Pearson correlation between a given week’s total COVID-19 deaths in a particular country and the following week’s enrollment rates from the first date a COVID-19 case was confirmed in that country up until the last date in our sample (March 28, 2022)

We then quantified the relationship between COVID-19 severity and industry-sponsored clinical trial screening rates outside of the USA. We repeated the measures we used to measure the US correlation for these two pandemic metrics in our global sample. One country of note with the most rapid return to pre-pandemic level clinical trial screening rate was the UK (see Figs. 1D and 4). The UK showed a significant, negative correlation between COVID-19 deaths and screening rate during both the first 12 weeks of the pandemic (*r* = − 0.74, *p* = 0.004) as well as during the entire pandemic (*r* = − 0.30, *p* = 0.001) (see Fig. 4). After the USA, the next four countries with the largest number of clinical trial protocols in our analysis are Spain, Italy, France, and Germany. We observe in each of these countries a statistically significant, negative correlation during the first 12 weeks of the pandemic (starting with the first week a COVID-19 case was confirmed in each country) between COVID-19 new death counts and clinical trial screening rate (see Fig. 5). Further, we observe in Spain and Italy a continued negative, significant correlation with screening rates from the onset of the pandemic to end of March 2022 (Spain *r* = − 0.19, *p* = 0.048; Italy *r* = − 0.19, *p* = 0.042), while France continues to have a marginally negative correlation (*r* = − 0.18, *p* = 0.059). These findings demonstrate in these initially profiled countries that COVID-19 severely impacted clinical trial screening rates during the first three months of the pandemic, while many countries continue to observe negative impacts as of the time of this writing.

**Fig. 6:**
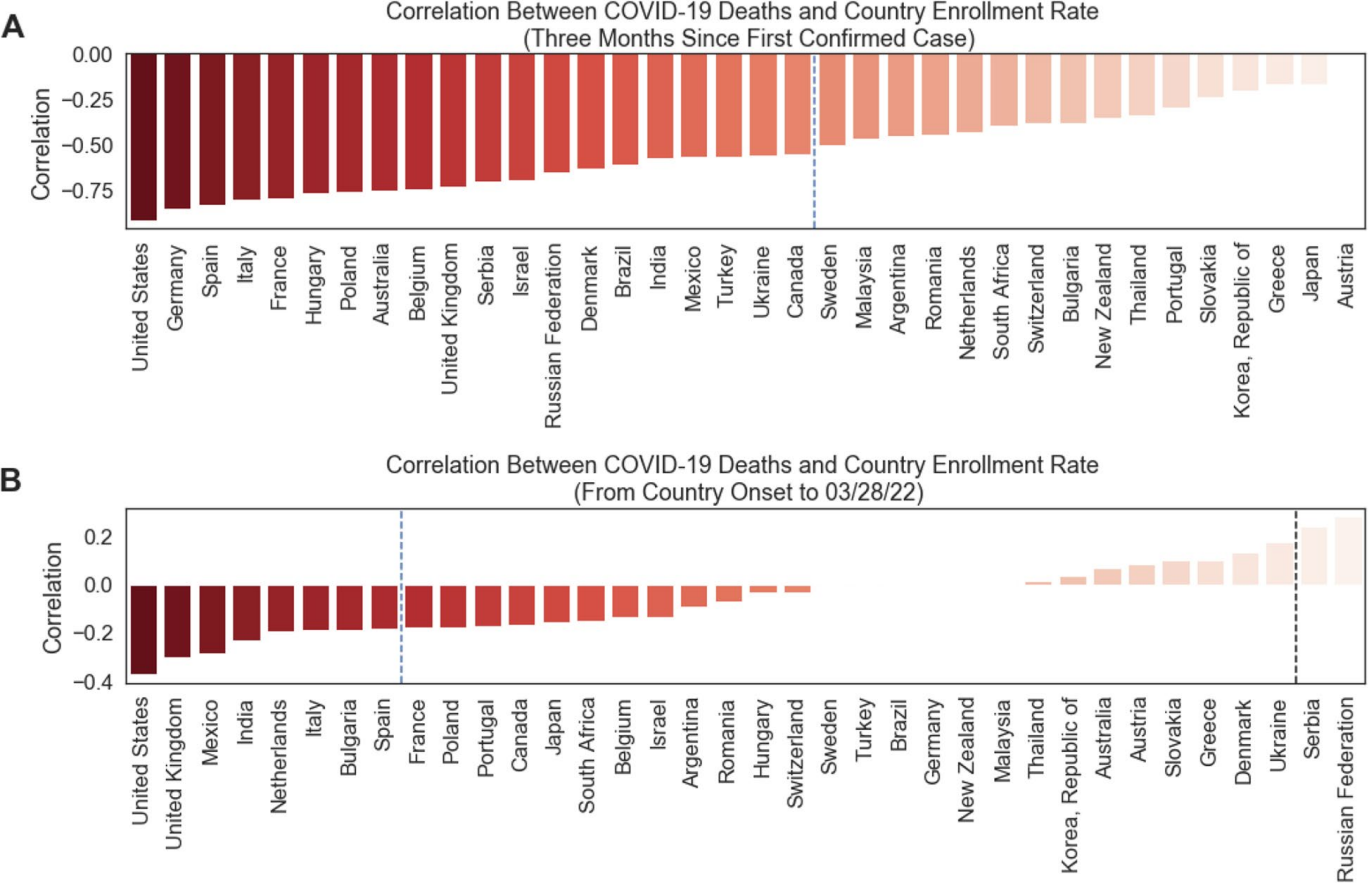
Comparative bar graph featuring each included country (in the top 50th percentile of number of unique trial protocols in our global clinical trial dataset) and its correlation between weekly number of COVID-19 deaths and the country’s clinical trial enrollment rates during the **A** first 12 weeks of the pandemic and **B** from the onset of the pandemic in each country until March 28, 22. The COVID-19 pandemic onset for a given country is defined as the week in which the first COVID-19 case was confirmed in that country. Bar color and bar height represent the magnitude of the Pearson correlation. Countries to the left of the dashed blue vertical line have a statistically significant, negative correlation between COVID-19 death count and trial enrollment rates, while countries to the right of the line have correlations that are not statistically significant. In **B**, we observe both Serbia and Russia as having significant, positive correlations, likely due to the ongoing Russia-Ukraine conflict. The negative correlations between COVID-19-related deaths and a country’s clinical trial enrollment rate were much stronger in the first 3 months of the onset of the pandemic compared to the correlation of the full time frame of the pandemic

Finally, we quantified the relationship between the onset of the pandemic and clinical trial screening rates for the remaining countries in our dataset with sufficient sample size. For this analysis, we include countries in both our proprietary dataset and in the Johns Hopkins COVID-19 dataset that are in the top 50th percentile of trial protocols in 2019 (at least 19 clinical trial protocols). We report in Fig. 6 the correlations between weekly COVID-19 death counts and that country’s clinical trial screening rate during the first 12 weeks of the pandemic. We observed, as predicted, a global, widespread trend of large, negative correlations between COVID-19 pandemic severity and clinical trial screening rates during the first three months of the pandemic. Every country included in the present analysis whose Pearson correlation was statistically significant at the *p* < 0.05 level was negative for the first three months of the pandemic, which was in line with our a priori hypothesis that a rise in COVID-19 severity in a particular country would lead to a decline in screening rates. In Fig. 6a, the dashed blue line separates the countries with a statistically significant (at *p* < 0.05 level) negative correlation between COVID-19 deaths and country screening rate on the left from the countries with non-significant correlations on the right.

We also observe that during the full duration of the COVID-19 pandemic up until end of March 2022, the correlations between COVID-19 deaths and clinical trial screening rates are less negative, with fewer countries reporting significant correlations between the two metrics. We also observe two countries, Serbia and Russia, that show positive, significant correlations, with Ukraine showing marginally significant correlations. Overall, while the decrease in clinical trial screening rates related to the pandemic is still being felt by many countries, we also observe a weakening of this disruption over time relative to the first three months of the pandemic.

## Discussion

In this study, we quantified the impact of the global COVID-19 pandemic on industry-sponsored clinical trial screening rates by examining the YOYD in screening rate by country and therapeutic area as well as analyzing the correlation between COVID-19 pandemic severity and screening rate both during the onset of the pandemic as well as over time. It verified the assumption that, overall, COVID-19 significantly and negatively impacted clinical trial screening and provided insights on the differential impacts the pandemic has had at a country level, therapeutic area level, and temporal level.

The impact of the pandemic was most extreme during the spring of 2020 when many countries first faced COVID-19 at a national scale. Given the lack of understanding of the virus in terms of severity, transmission, treatment, and prevention, coupled with a general societal concern, the impact on trial recruitment was part of the much larger lifestyle restrictions put into place by governments. Also, clinical trial sites either elected to not see any new trial participants until more information was known or their resources were diverted to urgent COVID-19-related care and research. While there was a massive decline in overall trial recruitment, some research activity continued such as in oncology where research represents part of cancer care and is reflected in our results.

We note in our analysis that, overall, the negative relationship between pandemic severity and clinical trial screening observed in more recent months has lessened compared to that of the first 12 weeks of the pandemic. This is in large part due to two major trends. First, the international scientific community, through tireless effort and unprecedented scholarly communication across the globe, has learned a great deal about COVID-19 and has created several vaccines to combat the spread of the virus [7]. A recent study estimated that vaccines approved for administration outside a clinical trial setting saved 14.4 million lives in 185 countries between December 2020 and December 2021 [8]. Second, society has grown tolerant and/or fatigued of the various social restrictions deployed almost universally in spring 2020 [9]. This hypothesis is supported in the analysis of the screening impact of the pandemic with a gradual lessening of the impact of the virus on screening rates even when infections and deaths were higher than in previous waves. For instance, the USA was impacted by multiple waves of viral variants throughout 2020 and 2021, and those waves had considerably higher death counts and total infections than in spring 2020. However, the decline in screening rates in clinical trials, though suppressed during those subsequent waves, was not as severe as in spring 2020. This may reflect also measures taken to adapt clinical research protocols to accommodate the pandemic, implementation of virus control measures [10], or a growing fatigue with viral containment protocols that also hinder business operations, particularly for commercial research sites.

Additionally, the country-level differences in pandemic-induced decline in trial screening are also observed. There have been many different national level responses to the pandemic, particularly from the perspective of social restrictions such as masking, public gatherings, full community lockdowns, and many others [11]. Some countries have deployed clear overarching, low-tolerance policies to aggressively contain the pandemic, whereas others have deployed varied approaches reflecting the state of the pandemic that have been followed by constituents. Others, such as the USA, have left those decisions to individual states and municipalities, creating a heterogeneous response spectrum within a single nation. Some countries have taken proactive measures to mitigate the lingering effects of the initial dip in clinical research activity following the onset of the pandemic, such as the Managed Recovery and the Research Reset programs in the UK (https://www.nihr.ac.uk/researchers/managing-research-recovery.htm).

Our results are broadly comparable to findings of other reports in the literature. An analysis of 321,218 non–COVID-19–related trials listed on the clinicaltrial.gov database showed that from January 2017 to May 2020, 28,672 (8.9%) trials were stopped (i.e., reported a switch in trial status from “recruiting” to “active and not recruiting,” “completed,” “suspended,” “terminated,” or “withdrawn”), revealing that during the initial months of COVID-19, an average of 1147 trials/month were stopped compared to an average rate of 638 trials/month in the pre-pandemic period, implying a significant slowdown in research activity [12]. Another study showed that between February and May 2020, the number of trials activated in the USA was just 57% of expected number [13]. Another study showed a 60% decrease in the number of launches of phase 1–4 oncology trials during January-May 2020 compared with the pre-pandemic period [14].

A notable limitation of this study is the fact that our data pertains mainly to industry sponsored studies (i.e., excludes academic studies). Additionally, our choice of inclusion/exclusion criteria limit the generalizability of our findings outside of the scope of our research. Furthermore, because our analyses were correlational, we cannot identify exact statistical causes for the negative impacts we observed surrounding the pandemic. Finally, these causes might differ between countries for a number of reasons, such as the heterogeneous global response to the pandemic.

For future work, we are continuing to monitor the impact of COVID-19 on clinical trial operations and understanding it from long-term perspective. We are also building predictive models that incorporate COVID-19 pandemic severity to forecast screening rate, which would allow us to further quantify the impact using model parameters and apply it towards business operations. These observations may assist future researchers with anticipating impacts of pandemics on the conduct of clinical trials on a global scale.

## Data Availability

Data sharing is not applicable to this article as no specifically defined datasets were generated or analyzed during the current study.
